# Supplementary material for: Towards using Tweet sentiment for infectious disease detection
Source: PLoS One. 2025 Jun 2;20(6):e0325166. doi: 10.1371/journal.pone.0325166 (PMC12129144; doi:10.1371/journal.pone.0325166)
Supplement: Attachment — Submitted filename: Response to Reviewers.pdf [file pone.0325166.s001.pdf]

Dear Reviewers,

We cordially thank you for your thorough feedback and comments. Your review has raised numerous major issues in the presentation and inaccuracies in the technical description that we have addressed. In addition to the revised manuscript, we are also attaching a version of the document that highlights our changes using blue color for newly added material and using ~~red color strikethrough~~ for parts that have been removed.

The following is a list of all reviewer comments (no comments omitted) followed by our immediate responses and an explanation of how we have addressed the comments in the revised manuscript.

Anonymous Reviewer 1:

Comment: *# Overview*

The paper aims to build on existing studies that find weak or mixed correlation between COVID incidence and Twitter sentiment in the United States by investigating the spatial and temporal variation in this correlation. To this end, the authors combine data from the TBCOV Tweet dataset with data on COVID prevalence from the CDC to generate average sentiment and COVID prevalence at the county–week level. They then calculate Pearson's correlation statistic on different subsets of this data.

Below, I give a high-level assessment of (a) the framing and literature review and (b) the methodology used. I then list some smaller issues that I encountered in the manuscript.

Comment: *1. # Framing and literature*

An initial issue with the manuscript is a mismatch between the framing and the analysis. The authors situate the importance of their analysis in terms of contributing to the rapid detection (or even prevention) of epidemic outbreaks. However the analysis itself does not provide any treatment of the the predictive value of sentiment for disease outbreaks. One of the main features of the relationship between COVID cases and Tweet sentiment illustrated in the paper is that there is a large amount of unexplained variability in this relationship across space and time. Because of this, the utility of the findings is overstated.

The literature that the authors cite at the beginning of the article does a good job motivating the potential for social media in epidemiological studies in general. However the paper overstates the novelty of the spatial analysis ti presents. Notably, the original paper accompanying the TBCOV dataset the paper relies on includes a similar county-level analysis.

I was also surprised that the authors did not reference the considerable body of research demonstrating systematic geographic variation in social media sentiment in the United States (e.g. Mitchell et al 2013). Given the emphasis on geographic variation in the analysis, such work seems particularly relevant.

Response:
Existing literature as it relates to the correlation between Tweet sentiment and COVID-19 is inconclusive, motivating our study. Similarly, our own results also show a large amount of spatial and temporal variation in the correlation, however we find that at some points in time and in some locations, these correlations are strong and significant. This indicates that Tweet sentiment could indeed be used as an indicator of COVID-19 prevalence and further research is needed to explore how this potential could be harnessed. We have pulled back on text that may appear overstated in the Discussion section.

Comment: *2. # Methodology*

Overall, the methodology used in the paper is well described, but it is overly simplistic for an investigation of the relationship between social media sentiment and disease prevalence. Among the main findings of the paper is that correlations between sentiment and COVID cases are often stronger when they are calculated on smaller geographically and temporally restricted slices of the data. This kind of finding should not be surprising, though: stronger correlations are exactly what one would expect if the data has any sort of underlying smoothness (i.e. temporal and spatial autocorrelation).

That said, the authors note some intriguing patterns such as the strong negative correlation from July to September of 2020 or the East–West differences in direction and clustering of correlation. Unfortunately, the authors do little to try to explain these patterns. Are the temporal (or geographic) patterns associated with national (or local) policy? Do the spatial patterns correspond with population density, socioeconomic differences, or medical resources? If the stated goal is to contribute to early warnings for outbreaks, explaining the differences in magnitude and direction of correlation is vital. In my view, a regression analysis that takes into account spatial and geographic features would be a much more appropriate method to use.

Response:

We run a linear regression (OLS) using the county level variables “percent population age 25+ with no high school education”, “primary care physicians per 100k population”, “percent unemployment”, “percent age 65+”, “percent of a population living in a rural area”, and “population density”. Overall the model does not explain the spatial patterns, with an adjusted R2 ranging from 0.2% for the overall period to 6% for wave one. However, we find that some of the variables are significant for certain periods. For example, in the first wave (March 02 2020 to June 07 2020), we find that as “percent of the population living in rural areas” of a county increases, the correlation between COVID-19 cases and sentiment becomes more negative (which is expected). We also find during this period that as “the number of primary care physicians” increase, the correlation between COVID-19 cases and sentiment becomes more positive (which is less expected).

We explain these spatial patterns in the correlations between COVID-19 cases and sentiment in a new section called "Explainations of Correlations". We discuss the implications of these findings in the Discussion section.

Comment: *3.*
- In Figure 1(b), the rolling 7-day average of COVID cases per 100K in Washington falls below zero at the beginning of 2021. This warrants explanation (a quick view of the cited COVID data source doesn't show any negative values)

Response: The CDC data that we are accessing via usafacts.org indeed has negative values for Washington State during the last weeks of December 2020 due to corrections and adjustments. The same data artefact can be found here:
https://usafacts.org/visualizations/coronavirus-covid-19-spread-map/state/washington/

Comment: *4. - On page 4, the authors state "Deriving the sentiment of each such set provides us with a function S(w, r) that returns the sentiment of all tweets observed in region r during week w." I couldn't find a description of how sentiment was aggregated across sets of tweets.*

Response: We use the the average sentiment (of a set of tweets). We've clarified this in the manuscript.

Comment: *5. - In figure 2(b) and in the text on pages 6 and 7 the authors use p-values in a loose way to assess significance. A statistic like a Z-score would be more appropriate.*

Response: Thank you for the suggestion, We have added an additional figure with the z-scores please see Figure 2(c).

Comment #6:
- On page 6, the authors state that "we observe that our hypothesis of temporal heterogeneity is supported," but relevant the hypotheses put forward on page 4 was that the correlation would be "stable over time."

Response: Thank you for catching this logical flaw! We've fixed this, and we now consistently formulate the hypothesis as spatial and temporal inhomogeneity on Page 5, and describe that this hypothesis is NOT supported by the data on Page 6.

Comment: *#7. - I do not believe the authors ever justified their choices of dates separating the three 'waves' used in the final spatiotemporal analysis.*

Response: Based on the correlation patterns observed in Figure 2, we divided our analysis into three distinct waves. The first wave corresponds to the period characterized by positive correlation coefficients, indicating sentiment moved in tandem with case numbers. The second wave is marked by predominantly negative correlation coefficients, suggesting an inverse relationship between sentiment and cases. The third wave represents the period when correlation coefficients returned to positive values. We focused our investigation on these three periods to understand the shifting dynamics between public sentiment and COVID-19 case numbers.

Anonymous Reviewer 2 ():

Comment: Towards Using Tweet Sentiment for Infectious Disease Detection
The authors explore whether social media sentiment, specifically derived from tweets, can function as an early warning system for infectious disease outbreaks, focusing on COVID-19. Utilizing a dataset of 56,755,894 tweets from the platform X, they analyze spatial, temporal, and spatiotemporal correlations between tweet sentiment and COVID-19 case counts, aggregating data by county and week. Their temporal analysis identifies a significant negative correlation between sentiment and cases from June to September 2020, suggesting that declining sentiment could predict outbreaks. Spatially, they find contrasting trends: a negative correlation in the Eastern U.S. and a positive correlation in the Western U.S., highlighting regional variability. Spatiotemporal analysis uncovers even stronger correlations during specific periods and regions, emphasizing the potential of social media as a complementary tool to traditional public health surveillance systems if contextual dynamics are better understood.
The paper is well-structured and employs a consistent methodology. However, there are several areas where the authors could improve their work before publication:

Comment: *1. Literature on Complex Systems and Social Media Communication*
The manuscript would benefit from incorporating insights from complex systems research that explore the relationship between social media communication patterns and disaster prediction, prevention or

even management after. For instance, studies like "Rapid Assessment of Disaster Damage Using Social Media Activity" could be relevant.

Response: We have reviewed the study "Rapid Assessment of Disaster Damage Using Social Media Activity" and similar literature to explore how social media communication patterns have been leveraged for disaster prediction, prevention, and management. We include a brief discussion of this in the Introduction and include the suggested reference.

Comment: *2.Critique of Traditional Data Collection Methods*
While the authors critique testing bias and the lack of standardized reporting metrics in traditional methods, additional points could strengthen this discussion. For example, official census data often relies on diverse methodologies and is influenced by budgetary constraints, while surveys and questionnaires can introduce subjectivity in evaluation criteria. In the specific context of COVID-19 mapping data, studies such as "Geospatial Analysis and Mapping Strategies for Fine-Grained and Detailed COVID-19 Data with GIS" highlight these issues and could provide a solid foundation to justify the approach taken in this study.

Response: We enhance our discussion in the Introduction about the limitations of reported case data, considering underascertainment of mild cases, time lags, budgetary constraints and limitations to resources, group characteristics, and issues in reporting and testing. We have added the suggested reference to our manuscript.

Comment: *3. Reliability of Social Media Data During Pandemics*
The authors argue that microblogs like tweets are effective in detecting disease prevalence (e.g., influenza, dengue). However, why did such globally accessible and rapid communication channels fall short during the COVID-19 pandemic? This question warrants exploration to address the limitations of social media in large-scale health crises.

Response: Communication channels like social media may have also fallen short during the COVID-19 pandemic because it became a source of misinformation and partianship. We discuss the limitations of social media in large-scale health crises in the Discussion section.

Comment: *4.Contradictory Results in the Literature*
The manuscript references findings like those by "Valdez et al were surprised to observe a positive correlation between US wide COVID-19 related Tweet sentiment and cases and deaths, meaning that as cases and deaths increase in the US, sentiment towards COVID-19 trends positive. This contradicts

what would intuitively be expected. In another example, Feng and Kirkley find a weak negative or absent correlation between state-level Tweet sentiment and COVID-19 and cases and deaths". How can these contradictory and counterintuitive results across studies be interpreted? A discussion of these inconsistencies in the literature would enhance the paper's depth.
**Response:** Our study shows that the relationship between sentiment and COVID-19 cases varies over space and time (and likely scale), explaining why we see some contradictory results in the literature. We add this point to our Discussion.<br><br>Studies show that sentiment may more directly tied to other external factors such as public health messaging, media coverage, and policy changes. These factors can influence sentiment in ways that are not directly tied to case counts or deaths but rather to public perceptions of government response or hope for recovery (e.g., sentiment increasing due to vaccine rollouts despite rising cases). We reiterate that our study focuses specifically on examining the relationship between sentiment and COVID-19 case trends to evaluate the potential utility of sentiment as a tool for early disease detection.

**Comment:** 5. - *Complexity of Spatial and Temporal Contexts*<br>*The authors should consider the temporal and spatial heterogeneity of the pandemic's impact. Policies and interventions varied significantly across counties and states, influencing social behavior and pandemic outcomes. For instance, Maleki (2022) examines social factors influencing compliance with COVID-19 interventions in the U.S., offering valuable insights into behavioral changes during the pandemic.*
**Response:** We agree with your statement. We added a new set of experiments that attempt to explain some of the patterns in the relationships between sentiment and COVID-19 cases (see our response to Reviewer 1, Comment 2). While we hypothesize that policies and interventions may be an important variable, such data is complex and fine grained dataset is not easily acquired. Future work may consider how policies and interventions influence these patterns.

**Comment:** 6. - *Matching Real-World and Social Media Populations*<br>*A key limitation of using Twitter data is the demographic bias of its user base, which tends to be younger, more educated, and urban. How well does this population represent broader societal trends? While some areas may align more closely with Twitter sentiment, others may diverge significantly. The authors should consider a twofold discussion:*<br>*(a) The replicability of real-world events in digital spaces, and*<br>*(b) The complexity of social space, where factors like heterogeneity, scale, and context introduce variability in data interpretation (e.g., Morales et al., 2022).*

Response: We agree that Twitter data has inherent biases in the user base. To our knowledge, authors of the TBCOV has not commented on the degree of bias in the data. However, numerous highly cited studies have been published that aim to quantify the bias in Twitter data more generally. They find that Twitter users with geolocated Tweets represent a small fraction of the population (1.15%) and that populous counties are overrepresented. They also find a bias towards male users and that over/under sampling of different races of users is a function of location. We incorporate a discussion of potential for such bias and how it could affect our interpretation of our results in the Discussion. In response to your comment a) we incorporate a discussion about how high profile events or media driven narratives may disproportionately shape sentiment on Twitter, potentially diverging from broader public sentiment and b) we incorporate a discussion about how variability in factors like regional cultural norms, socio-political climates, and local pandemic responses can lead to divergent sentiment patterns. We also highlight how differences in spatial scales—such as local versus national—can affect the interpretation of sentiment trends.

Comment: 7. - *Expanding Discussion and Results*
The discussion and results sections are currently underdeveloped. While the authors present key findings, they provide limited analysis or interpretation. Expanding these sections with a deeper examination of results, including comparisons with previous studies and a critical evaluation of implications, would significantly enhance the paper's impact.

Response: Review 1 had a similar comment (see Comment 2, primarily). We expanded both our experiments as well as our examination of the results and discussion. We thank both the reviewers for the opportunity to improve our manuscript.
